# Neonatal Tactile Stimulations Affect Genetic Generalized Epilepsy and Comorbid Depression-Like Behaviors

**DOI:** 10.3389/fnbeh.2020.00132

**Published:** 2020-07-23

**Authors:** Aymen Balikci, Gul Ilbay, Nurbay Ates

**Affiliations:** Department of Physiology, Faculty of Medicine, Kocaeli University, Kocaeli, Turkey

**Keywords:** absence epilepsy, depression, handling, maternal separation, neonatal tactile stimulation, WAG/Rij rat

## Abstract

Recent studies suggest that development of absence epilepsy and comorbid depression might be prevented by increased maternal care of the offspring, in which tactile stimulation induced by licking/grooming and non-nutritive contact seem to be crucial. In this study, we aimed to evaluate the effect of neonatal tactile stimulations (NTS) on absence epilepsy and depression-like behaviors in adulthood. Wistar Albino Glaxo from Rijswijk (WAG/Rij) rat pups with a genetic predisposition to absence epilepsy were divided into tactile stimulation (TS) group, deep touch pressure (DTP) group, maternal separation (MS) group or control group. Between postnatal day 3 and 21, manipulations (TS, DTP, and MS) were carried out for 15 min and three times a day. Animals were submitted to locomotor activity, sucrose consumption test (SCT) and forced swimming test (FST) at five months of age. At the age of six months, the electroencephalogram (EEG) recordings were conducted in order to quantify the spike-wave discharges (SWDs), which is the hallmark of absence epilepsy. The TS and DTP groups showed less and shorter SWDs in later life in comparison to maternally separated and control rats. SWDs’ number and total duration were significantly reduced in TS and DTP groups whereas mean duration of SWDs was reduced only in DTP group (*p* < 0.05). TS and DTP also decreased depression-like behaviors measured by SCT and FST in adult animals. In the SCT, number of approaches was significantly higher in TS and DTP groups than the maternally separated and control rats. In the FST, while the immobility latency of TS and DTP groups was significantly higher, only TS group showed significantly decreased immobility and increased swimming time. The results showed that NTS decreases both the number and length of SWDs and the depression-like behaviors in WAG/Rij rats probably by increasing arousal level and causing alterations in the level of some neurotrophic factors as well as in functions of the neural plasticity in the developing rat’s brain.

## Introduction

Epilepsy is a common neurological condition that effects nearly 1% of the population (Bailey and Im-Bolter, 2018). Despite the introduction of many new antiepileptic drugs, 30% of epileptic patients are resistant to antiepileptic drugs (Nazish et al., 2018). Furthermore, drugs that are currently used to treat epileptic symptoms have not been found clinically effective against epileptogenesis (Citraro et al., 2017). The Wistar Albino Glaxo from Rijswijk (WAG/Rij) rat strain, as well as being a widely used genetic model of generalized absence epilepsy, is also a potential animal model to investigate epileptogenesis (Coenen and van Luijtelaar, 1987; White and Löscher, 2014). Spike-wave discharges (SWDs) in the electroencephalogram (EEG) start to appear in 2–3-month-old WAG/Rij rats. Behavioral arrest, twitching of vibrissae, staring, accelerated breathing and head tilting are behaviors that accompany EEG seizure activity (van Luijtelaar and Sitnikova, 2006; van Luijtelaar and Zobeiri, 2014). Around 6 months of age, animals of this strain generally show 16–20 SWDs an hour (Schridde and van Luijtelaar, 2004). Recently, WAG/Rij rats have been validated as a model for mild depression (Russo et al., 2011; Sarkisova and van Luijtelaar, 2011). It has been suggested that seizures are required for the emergence of depression-like behavior (Sarkisova et al., 2010).

Although of strong inherited component, absence epilepsy and comorbid depression-like behaviors in WAG/Rij rats are influenced by environmental and epigenetic factors and are quite sensitive to early interventions (Schridde et al., 2006; Sarkisova and Gabova, 2018; Smirnov et al., 2018). For example, in WAG/Rjj rats, early neonatal handling results in a decrease of epileptic activity at adult ages (Schridde et al., 2006). Whisker trimming during postnatal period in WAG/Rjj rats also changes seizure activity later in life (Sitnikova, 2011). Blumenfeld et al. (2008) demonstrated that early and chronic administration of the anti-absence drug ethosuximide suppressed the development of SWD in WAG/Rij rats. Moreover, recent studies showed how maternal care (MC) during the neonatal period affects epileptogenesis in WAG/Rij rats. Symptomatic WAG/Rij mothers showed poorer maternal care when compared to aged matched Wistar controls (Dobryakova et al., 2008; Sitnikova et al., 2016).

Sitnikova et al. (2015) demonstrated that WAG/Rij rat pups nursed by Wistar mothers showed less seizure activity later in life. Sarkisova et al. (2017) showed that increased MC prevented the absence epilepsy development and comorbid depression in the same model (Sarkisova et al., 2017). Taking all this into consideration, poor maternal care in WAG/Rij rats might negatively alter rat pups’ early brain development, promoting absence seizures in adulthood.

Maternal behavior in rats includes nursing, collecting pups, crouching over the pups or arched-back nursing, licking and grooming (LG) (Macbeth et al., 2010). Mother-pup interaction studies have mostly pointed on the role of LG and arched-back nursing (ABN) (Caldji et al., 2003; Kaffman and Meaney, 2007; Daskalakis et al., 2012). Studies generally accept that ABN is a more favorable position for feeding when compared to non-arched-back nursing (NABN) (Lonstein et al., 1998). However, Sarkisova and Gabova (2018) suggested that non-nutritive contact with pups is also an important aspect of maternal care, which improves pathologic phenotype of WAG/Rij pups later in life. Another study showed that a high level of non-nutritional nursing normalizes the pathologic phenotype of rats with schizophrenia (van Vugt et al., 2014). Both maternal LG and NABN are considered as particularly influential stimuli and as essential sources of tactile sensory stimulation for pups, crucial for the maturation of the nervous system (Gonzalez and Fleming, 2002; Melo, 2015; Sarkisova and Gabova, 2018).

TS is a method of sensory stimulation that resembles maternal LG behavior in rats. Neonatal TS enriches the experience and improves maturation of newborn animals, positively influencing behaviors and neuroendocrine systems in pups (Boufleur et al., 2012, 2013; Antoniazzi et al., 2014). Others showed that neonatal TS increases neurogenesis and neuroplasticity, improves anxiety-like behaviors and prevents depression-like behaviors (Richards et al., 2012; Freitas et al., 2015; Roversi et al., 2019). Recent reports have shown that when TS is applied both during the initial periods of development and in adult rats, neurotrophins such as brain-derived neurotrophic factor (BDNF) and fibroblast growth factor 2 (FGF-2) increase in different brain areas. In addition, dendritic lengths after brain injury increase and memory performance enhances (Roversi et al., 2019).

Considering the effect of maternal care on incidence of absence seizures in WAG/Rij rats and the promising benefits of neonatal TS during the initial periods of development, we investigated the effects of two distinct forms of neonatal TS, light TS (stimulus that mimics LG) and deep touch pressure (DTP; stimulus considered could have a potential of mimicking NABN) on absence epilepsy and comorbid depression-like behaviors in adult WAG/Rij rats.

## Materials and Methods

### Animals and Experimental Procedure

All procedures were performed in accordance with welfare guidelines approved by Ethics Committee of the University of Kocaeli, Turkey (KOU-6/5-2016).

Pregnant female WAG/Rij rats from the breeding facility of University of Kocaeli were housed individually in plexiglas cages in a temperature controlled room at 22–23°C and on a 12:12 h light:dark cycle. The births were monitored and at postnatal day one (P1) rat pups were randomly divided into one of four groups for each litter: control (C), maternal separation (MS), light tactile stimulation (TS) and deep touch pressure (DTP).

**FIGURE 1:**
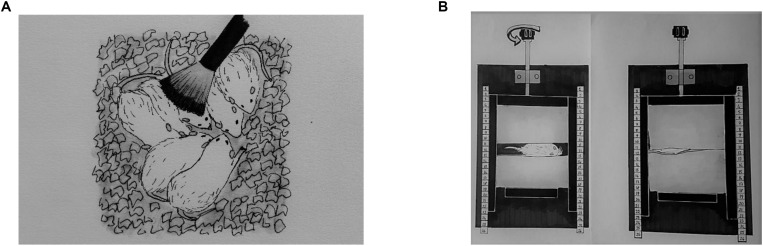
A representative illustration of neonatal tactile stimulations. Light tactile stimulation **(A)**, deep touch pressure **(B)**.

Light TS, DTP, and MS were applied three times per day at 9:00 AM, 1:00 PM, and 4:00 PM from P3 to P21. Mothers and their pups at the control group stayed in the home cage while TS, DTP, and MS group pups were removed from the home cage for each session. Next a soft baby brush was used to brush rat pups in the TS group during 15 successive minutes (Figure 1A). The TS protocol was adapted from Mychasiuk et al. (2013).

The DTP apparatus was adapted from Grandin (1992); it was made of wood and was composed of one “]” shaped mobile wall (lateral walls: 20 × 5 cm, middle wall: 10 × 5 cm; one of lateral wall has a window for observation of the pup) that was moved with an adjustable compression lever, one stable wall (10 × 5 cm) that fits inside the “]” shaped mobile wall, a 30 × 20 cm base with two support bars (30 × 2 cm) for the mobile wall and two sponge pads inside the walls (22 dns, 5 × 10 cm). Pups were placed individually between pads facing the window of mobile wall. To ensure equal delivery of pressure, the pups were placed between the sponge pads that were close enough to touch each other with the adjustment lever (Figure 1B). Next, the pups returned to their litter.

In MS, pups were placed in a separate warmed cage for 15 min three times a day at 9:00 AM, 1:00 PM, and 4:00 PM between P3–P21. All rat pups were placed back with their mother after each session. Rat pups in the control group were not given any stimulation and were only handled during the regular home cage cleaning, two times a week. Following TS, DTP, and MS on P21, pups were weaned and housed with their same sex siblings in groups of 10.

### Behavioral Evaluations

At the age 5 months, all male rats were tested in a calm and dimly lit environment (stress-free conditions) between 10.00 am and 3.00 pm. Test sequence for each animal was performed as first locomotor activity, next sucrose consumption test and these were followed by the forced swimming test.

#### Locomotor Activity Test

Rat activity monitoring system, comprised of a plexiglas chamber (42 × 42 × 30 cm), a computer, and open field activity software, was used to record locomotor activity (Commat Ltd., Ankara, Turkey). After accommodation to the room for 1 h, rats were placed in the plexiglas chamber and locomotor activity was recorded as interruptions of photocell beams for 10 min by the software. Total locomotor activity (vertical, ambulatory and stereotypic activities) was evaluated (Karson et al., 2012).

#### Sucrose Consumption Test

Each rat was placed in separate cages, the intake of 20% sucrose solution and the number of approaches to the drinking bottle were measured for 15 min (Sarkisova and Kulikov, 2006). By weighing the bottle in the beginning of and at the end of the test, sucrose intake was calculated. Before testing, animals were not kept without food or water. After adaptation to the procedure for 3 days, sucrose consumption values on the 4th day were made use of for the comparison of differences between rat groups. The measurement of sucrose consumption was used to assess animals’ anhedonic-like states and the number of approaches was used as indirect indicator of explorative activity during the test.

#### Forced Swimming Test

The forced swimming test was carried out in a cylinder-shaped test apparatus (height, 47 cm; inside diameter, 38 cm) filled with 38 cm of tap water at 22 ± 1°C. Firstly, for conditioning, rats were placed into the cylinder for 15 min. Then, having been removed from the cylinder, rats were gently dried, warmed and were put back to their home cages. 24 h later, rats were placed back into the cylinder for an experiment swimming session for 5 min. Following the aforementioned session, the drying and heating procedure was repeated again.

Tests were videotaped and the observer blind to the treatment conditions measured the duration of immobility, the immobility latencies and the duration of active swimming. Vertical swimming necessary to keep only the head above the surface of the water was defined as passive swimming or immobility (Cryan and Lucki, 2000). In this test, the indications of depressive-like behaviors are decreased immobility latencies, increased immobility duration and decreased active behaviors.

### Surgery and EEG Recordings

**FIGURE 2:**
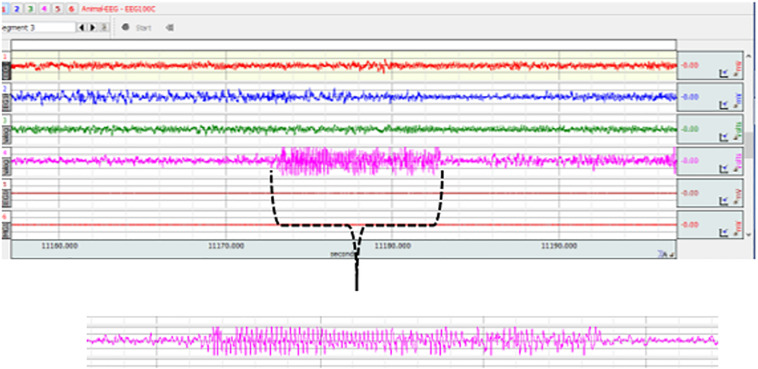
Representative EEG recordings from TS (1), DTP (2), MS (3) and control (4) rats. 4th trace and expanded view shows a typical 7–10 Hz SWD lasting for approximately 10 s.

At the age of 6 months, 8 animal per group were implanted with electrodes for EEG recordings. Cortical tripolar electrode set (MS3333/2A; Plastic One, United States) was placed with stereotaxic surgery under complete Xylazin (5 mg/kg ip) and Ketamine (60 mg/kg ip) anesthesia. The first electrodes were placed in the frontal area (AP 2.0 mm, L 3.5 mm), the second in the parietal area (AP -6.0 mm L 4.0 mm) at the surface of cortex and the reference electrodes were placed in the cerebellum (Ates et al., 2004). After surgery, the animals were kept in separate cages and were allowed to recover for 2 weeks. For EEG records, subjects were put in recording cages and connected to a computerized EEG recording system (MP150, Biopac Systems Inc., Santa Barbara, CA, United States) with a flexible cable that allows to move. After the 1-h habituation of the rats to the recording conditions, the EEG recordings were conducted between 10.00 am–2.00 pm for 4 h. SWDs were assessed using fundamental criteria: SWDs which occur between 1 and 10 s, with sharp spikes of frequencies between 7–10 Hz and asymmetrical spike amplitudes with slow waves and double background activity (van Luijtelaar and Coenen, 1986; Ovchinnikov et al., 2010). Number, mean duration and total duration of SWDs per hour were determined (Figure 2).

### Statistical Analyses

All statistical analyses were performed in GraphPad Prism 7.03 (United States) statistics program using one-way ANOVA or two-way ANOVA, followed by *post hoc* Tukey’s test when appropriate. Groups (4 levels) were always used as a between subjects factor. For the analyses of SWDs hour was used as a within subjects factor. A *p*-value less than 0.05 was considered to be statistically significant. Data are shown as mean ± SEM.

## Results

### Behavioral Tests

**FIGURE 3:**
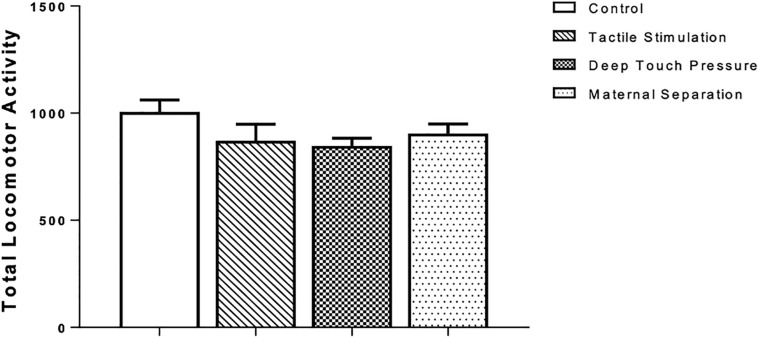
Locomotor activity in WAG/Rij rats submitted to TS, DTP, and MS in comparison to unhandled control group. The data are presented as mean ± SEM (*n* = 10 per group). Total locomotor activity, photocell count in 10 min (*P* > 0.05). There was no statistically significant difference among groups according to one-way ANOVA.

There were no significant differences in the total locomotor activity, as revealed by one-way ANOVA, between intervened and control WAG/Rij rats (Figure 3).

**FIGURE 4:**
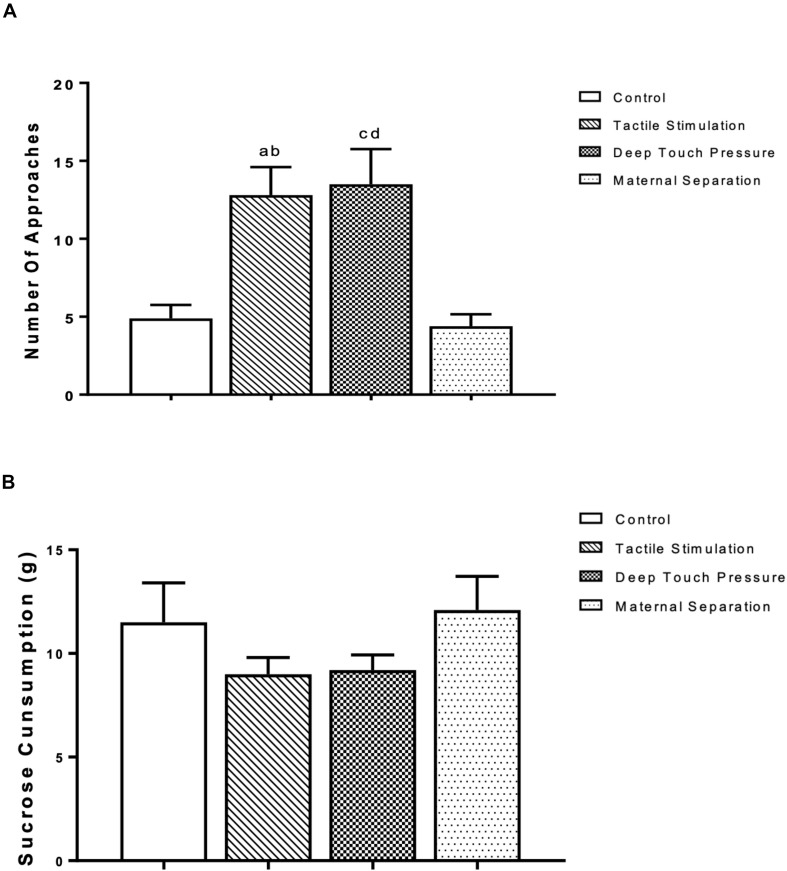
The mean ± SEM (*n* = 10 per group) for the amount of sucrose consumed. The influence of TS, DTP, and MS on behavioral parameters in the sucrose consumption test. **(A)** Number of approaches to the bottle; TS compared to the control (^a^*p* < 0.001) and MS groups (^b^*p* < 0.005); DTP compared to the control (^c^*p* < 0.001) and MS groups (^d^*p* < 0.005). **(B)** There was no statistically significant difference in the amount of sucrose consumption among groups according to one-way ANOVA measures (*P* > 0.05).

The outcomes of the sucrose consumption test are presented in Figure 4. The one-way ANOVA revealed significantly higher (*p* < 0.001 and 0.005) number of approaches in TS group compared to control and MS groups, and in the DTP group in comparison with control and MS groups. There were no significant differences in sucrose consumption between TS, DTP, MS and control groups according to one-way ANOVA (*P* > 0.05).

**FIGURE 5:**
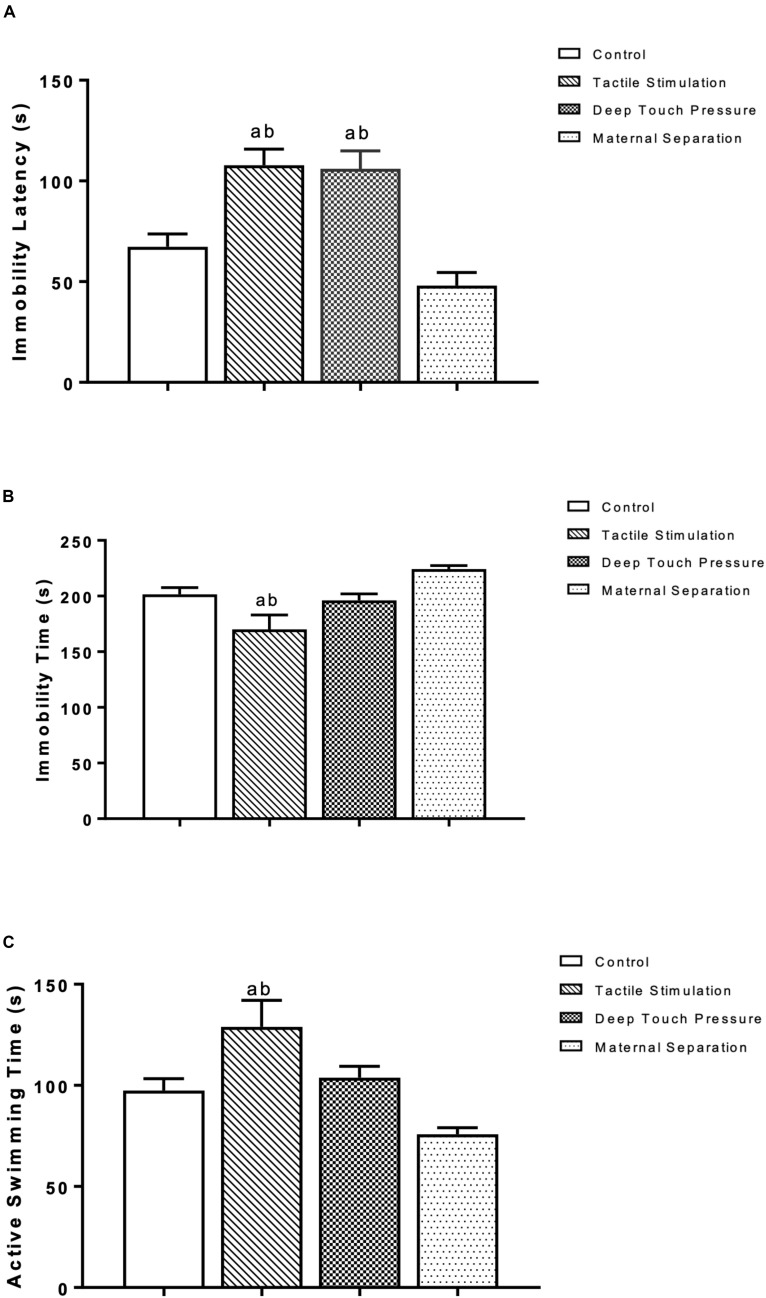
Behavioral measures at the forced swimming test in WAG/Rij rats. The data are presented as mean ± SEM (*n* = 10 per group). Immobility latency at **(A)** TS and DTP groups compared to the control group (^a^*p* < 0.005) and immobility latency at TS and DTP groups compared to the MS group (^b^*p* < 0.001); **(B)** Immobility duration, TS group compared to the control group (^a^*p* < 0.05), TS group compared to MS group (^b^*p* < 0.001); **(C)** Active swimming duration, TS group compared with control group (^a^*p* < 0.05), TS group compared with MS group (^b^*p* < 0.001) (one-way ANOVA).

The latency to immobility in the forced swimming test of the TS and DTP groups was significantly higher than control and MS groups (*p* < 0.005 and 0.001). The TS group demonstrated decreased immobility duration and increased active swimming time compared to the control and MS groups (one-way ANOVA, *p* < 0.05 and 0.001). The data are presented in Figure 5.

### SWDs

**FIGURE 6:**
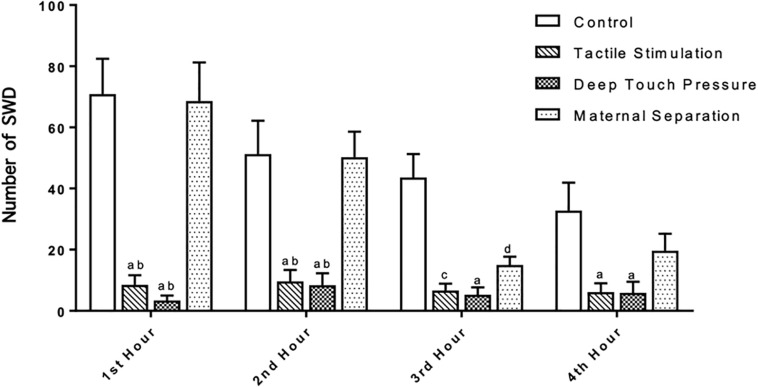
Neonatal TS and DTP influences the number of SWDs in adult WAG/Rij rats. The data are presented as mean ± SEM (*n* = 8 per group). Rats underwent TS and DTP showed less SWD in their EEG at 1st and 2nd hour of recordings compared to control (^a^*p* < 0.001) and MS groups (^b^*p* < 0.001). TS (^c^*p* < 0.005), DTP (^a^*p* < 0.001), and MS (^d^*p* < 0.05) groups showed less SWD than control group at 3rd hour and number of SWD was less in TS and DTP (^a^*p* < 0.05) groups at 4th hour of recordings (two-way ANOVA).

The number, mean duration and total SWD time were calculated in control, TS, DTP, and MS WAG/Rij male rats per hour. SWD met the criteria provided by van Luijtelaar and Coenen (1986). TS and DTP groups showed very few SWD. SWDs were not detected in 4 out of 8 animals in the DTP group in any of four hours. Two-way ANOVA performed for SWD number [F(9,112) = 3.533, *p* = 0.0007] showed a significant interaction between the TS and DTP effects. TS and DTP decreased significantly the number of SWDs in first two hours compared to control and MS WAG/Rij rats with two-way ANOVA (*p* < 0.001 and <0.00). The number of SWDs was significantly lower in TS, DTP, and MS WAG/Rij rats compared to the control group at the third hour (*p* < 0.001, 0.005, and 0.05) and significantly lower in TS and DTP group in fourth hour compared to control WAG/Rij rats (*p* < 0.05) (Figure 6).

**FIGURE 7:**
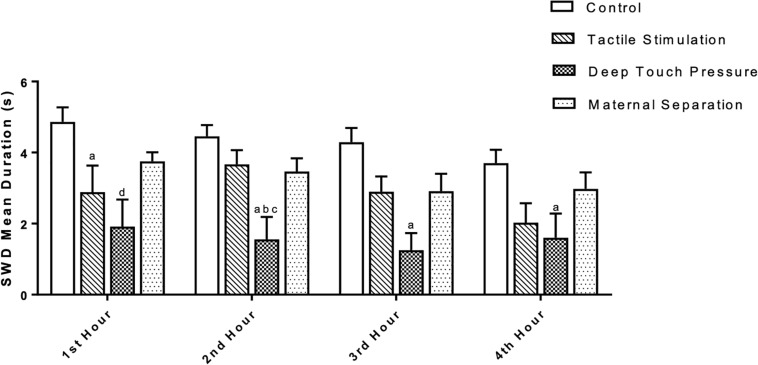
The mean duration (in seconds) of SWD was different between groups. The data are presented as mean ± SEM (*n* = 8 per group). Mean SWD duration in TS (^a^*p* < 0.05) and DTP (^d^*p* < 0.001) groups was less compared to control group at the 1st hour. At the 2nd hour of recordings mean duration of SWD was less at DTP group compared to control (^a^*p* < 0.005), MS (^b^*p* < 0.05), and TS (^c^*p* < 0.05) groups. Mean duration of SWD was less in DTP group at 3rd (^a^*p* < 0.001) and 4th (^a^*p* < 0.05) hours compared to control group (two-way ANOVA).

**FIGURE 8:**
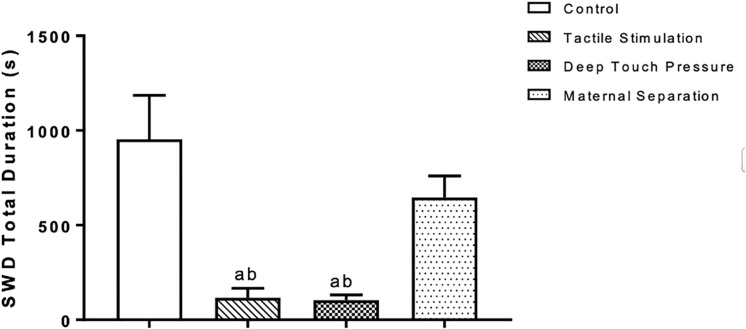
Mean and SEM (*n* = 8 per group) of the total duration of SWD was less in TS and DTP groups compared to control (^a^*p* < 0.005) and MS (^b^*p* < 0.05) groups at the four-hour recording period (one-way ANOVA).

Two-way ANOVA performed for SWD mean duration [F (3,112) = 20.23, *p* = 0.0001] showed a significant TS and DTP effects for the 1st hour and DTP effects for the remaining time period. Mean SWD duration in both TS (^a^*p* < 0.05) and DTP (^d^*p* < 0.001) groups was decreased at the 1st hour of recordings. DTP group showed statistically significant decrease of mean duration of SWD at the 2nd hour of recordings compared to control (^a^*p* < 0.005), MS (^b^*p* < 0.05) and TS (^c^*p* < 0.05) groups. Mean duration of SWD was less in DTP group at 3rd (^a^*p* < 0.001) and 4th (^a^*p* < 0.05) hours compared to control group (two-way ANOVA) (Figure 7). Total duration of SWD was less in TS and DTP groups compared to control (^a^*p* < 0.005) and MS (^b^*p* < 0.05) groups at four-hour period (one-way ANOVA) (Figure 8).

## Discussion

The main outcome of our study is that experimentally induced neonatal tactile stimulations can influence absence seizures and comorbid depression-like behaviors of WAG/Rij rats in adulthood. Particularly, our results clearly indicate that both neonatal TS and DTP between postnatal 3–21 days led at adulthood to: (i) a prominent reduction in SWDs and (ii) decreased depression-like behavior. More specifically, neonatal induced TS and DTP resulted in a lower number and shorter duration of SWDs than was seen in control and maternally separated WAG/Rij rats. Both neonatal TS and DTP induced higher immobility latencies in the FST while only TS increased swimming time and decreased immobility time compared to control and MS rats. TS and DTP showed increasing effect on frequency of approaches to the bottle in the sucrose consumption test while total consumed sucrose was not changed.

This is the first report showing that both TS and DTP might prevent the development of absence epilepsy and comorbid depression at genetically determined absence epilepsy. Differences in maternal behavior expression can provide various levels of somatosensory experiences for offspring in the first years of life, and these experiences have the potential to affect neurodevelopment (Fish et al., 2004). Sarkisova and Gabova, 2018 have recently found that WAG/Rij pups raised by Wistar dams exhibited less and shorter absence seizures which were accompanied by decreased depression-like behaviors in adult age compared to WAG/Rij pups raised by their WAG/Rij mothers that have decreased maternal care level. Therefore, it has eventually been suggested that improving maternal care in rats with genetic predisposition to absence epilepsy may be a way of reducing epileptic activity and depression-like comorbidity in adulthood.

Our results are in line with previous research findings that suggest environmental experiences early in life are important for the development of genetically predetermined absence epilepsy (Giblin and Blumenfeld, 2010; Sitnikova et al., 2015). Schridde et al. (2006) found that WAG/Rij pups that were exposed to daily handling between postnatal day 1 through day 22 showed a reduction in the number of SWD in adulthood; others showed that separation of pups from their mothers for 180 min during this timeline resulted in the same effect on SWD number (Sitnikova, 2011). It is suggested that decreased number of SWDs is related to the increased licking behavior (which is compensatory) by the dams to the pups after the MS (Czarnabay et al., 2019). In our study, MS did not induce any alteration in behavior and seizure activity. These results seem to be related to the short MS sessions as it has been demonstrated that prolonged separation periods that were more than 1 h early in life resulted in animals with behavioral and neuroendocrine signs of elevated stress in adulthood (Aisa et al., 2007).

A recent cross-fostering study has also found that when WAG/Rij pups were raised by Wistar mothers, the frequency and duration of SWDs were decreased compared with WAG/Rij rats raised by their own mothers (Sitnikova et al., 2015).

Maternal care in rats includes, besides nursing, two primary behavior types: (i) LG (a form of sensory manipulation to the skin) of pups and (ii) NABN (non-nutritive contacts with pups) (Richards et al., 2012). It has been shown that LG behavior of mother rats can be mimicked by TS. We consider that DTP could be a form of somatosensory stimulation that mimics NABN. Somatosensory stimulation including the use of the whiskers is a major source of sensory information in rodents, and the presence of a focal area in the peri-oral region of WAG/Rij rats with an increased neural excitability is typical for the WAG/Rij and also for the Genetic Absence Epilepsy Rats from Strasbourg model (van Luijtelaar and Zobeiri, 2014).

Present study findings showed that TS or DTP that mimics natural factors such as maternal care during early period could be capable of suppressing development of SWDs, perhaps by decreasing the heightened neural excitability and diminished GABAergic inhibition in the somatosensory cortex and the cortico-thalamo-cortical circuits involved in the occurrence and maintenance of SWDs. It should be noticed that TS and DTP exert a substantial effect on number and duration of SWDs in adult rats, showing the importance of level of experienced touch sensations during development. On the other hand, while both number and duration of SWDs were reduced at the TS and DTP exposed rats, only DTP showed reducing effect on mean duration of SWDs at four-hour time window. Interestingly, the mean duration of SWDs in WAG/Rij rats is primarily determined by the GABAergic reticular thalamic nucleus (Lüttjohann and van Luijtelaar, 2015). These findings point that early TS and DTP exposure induced changes in genetically determined SWDs in a stimulation-specific way during an early developmental period in cortex and thalamus, perhaps in other brain regions such as hippocampus, amygdala, hypothalamus and brainstem as well (Onat et al., 2013), since the effects of epileptogenesis and antiepileptogenesis are not limited to the focal zone and affect other brain areas outside the for SWD crucial cortico-thalamo-cortical loop.

There is a clear circadian variation at the distribution of the SWD activity with a maximum during the early hours of the dark phase and a minimum at the beginning of the light phase, when rats have the highest amount of deep slow-wave sleep (van Luijtelaar and Coenen, 1988; Drinkenburg et al., 1991; van Luijtelaar et al., 2001). Similar to previous studies, the effects of vigilance states on 7–11 Hz SWD distribution have been observed (Smyk et al., 2010). While all rats had the highest SWD amount during the first hour of the recording, they showed the minimum occurrence of SWDs during the 4th hour. Therefore, it is likely that the occurrence of SWD is facilitated when the arousal level is lowest and is suppressed when the arousal level is highest, which might be induced in NTS exposed rat groups that showed more active swimming and less immobility time.

It is also indicated that while a molecular mechanism in the thalamo-cortical circuit controls the occurrence of SWD, the other mechanism controls the duration of these discharges (van Luijtelaar and Coenen, 1988). In our study, it is clear that the reduction seen in the total duration of the SWD were originated from the decreased number of SWD occurrence due to TS and DTP induced mobility and arousal level.

It is shown that densities of GABAergic interneurons in hypothalamus and amygdala, brain regions sensitive to stressful stimuli and involved in the response to these stimuli, were increased by neonatal handling (Giachino et al., 2007). The assumed deficient cortical GABAergic system is sensitive to early environmental manipulations. Moreover, alteration of GABA_A_ receptor density related to increased maternal care levels was also shown in locus coeruleus (Caldji et al., 2003). Adult offspring reared by high licking and grooming mothers have significantly higher GABA_A_ receptor binding levels, which shows that NTS produces persistent changes in the GABA_A_ receptor complex of rats (Caldji et al., 2000; Raza et al., 2015).

NTS provided by LG and NABN during maternal care also can affect the offspring’s BDNF levels and neural development. BDNF has an essential role on the structural and functional plasticity in the brain (Lu, 2003; Lu et al., 2014; Leal et al., 2015). When BDNF is released from corticostriatal neurons and is bound to the tyrosin kinase tropomyosin-related kinase B receptors, multiple intracellular pathways are activated, as a result of which neurite growth, synaptic plasticity, proliferation, and survival are controlled (Castrén and Rantamäki, 2010; Yoshii and Constantine-Paton, 2010). It should be emphasized that BDNF is involved in the maturation of inhibitory GABAergic synapses (Henneberger et al., 2002; Yamada et al., 2003; Berghuis et al., 2004; Zhu et al., 2019), as well as that maturation and functioning of excitatory and inhibitory transmission is also modulated differently by BDNF (Gottmann et al., 2009; Meis et al., 2019). Moreover, it has been shown that early NTS treatment leads to increase in the skin and the brain production of FGF-2 (Richards et al., 2012). Studies suggested that increased FGF-2 following TS contribute to brain plasticity in animals (Comeau et al., 2007; Gibb et al., 2010; Richards et al., 2012).

NTS is considered as a positive early life experience that reorganizes neuronal morphology and synaptic connectivity during brain development (Kolb and Gibb, 2010; Richards et al., 2012; Mychasiuk et al., 2013; Raza et al., 2015). In a previous experiment, with the aid of Golgi technique, the dendritic features of cortical neurons, which might contribute to the increased cortical focal excitability have been revealed (Karpova et al., 2005).

Our findings also indicate that tactile experiences are an important modulator on depression -like behaviors of WAG/Rij rats. First, BDNF is involved in the origin of depression and in antidepressant effects and NTS enhanced BDNF (Martinowich et al., 2007; Antoniazzi et al., 2017). Next, NTS decreased depression-like behavior later in life (Freitas et al., 2015): TS early in life was accompanied by an increase in active swimming time and a decrease in immobility time in FST. Handling in animals resulted in different behavioral, endocrinological and neurochemical adaptive responses, suggesting the involvement of various neural pathways. During postnatal week 2, NTS was applied for the prevention of the development of neuropsychiatric disorders and it has been shown that NTS has modulating effect on hypothalamic-pituitary-adrenal (HPA) axis response later in life (Jutapakdeegul et al., 2003; Antoniazzi et al., 2014; Freitas et al., 2015). It can be hypothesized that NTS early in development counteracts the expression of depression-like behaviors of adult WAG/Rij rats by modulating HPA axis response and 5-hydroxitryptamine (5-HT) tone.

It is important to note that TS administration in both neonatal and adulthood causes increased trophic factor levels (BDNF and FGF-2) in different brain areas. The maturation and survival of neurons and (Lipsky and Marini, 2007; Antoniazzi et al., 2017) 5-HT_2A_ receptor functioning that is involved in the antidepressant activity are related to essential role of BDNF signaling (Tsybko et al., 2014; Popova et al., 2017; Roversi et al., 2019). In this sense, it is suggested that the influence caused by TS could improve HPA axis signaling and enhance the effects of neurotrophic factors which could consequently bring along improvement in depression-like behaviors in adult rats (Roversi et al., 2019).

On the other hand, Sarkisova et al. (2010) found that chronic treatment of WAG/Rij rats with ethosuximide in early life has a suppressing effect on the occurrence and repetition of seizures, and which also caused an antidepressant-like effect. They suggest that depression-like behaviors in WAG/Rij rats are provoked by occurrence and repetition of seizure activity. Recent data also indicate that treatment of WAG/Rij rats before the onset of the seizures with anti-absence therapy has the potential to reduce or delay the occurrence of seizures. Considering the remarkable effect of both forms of NTS on absence seizures, the symptoms of depression behaviors could be suppressed by reducing seizure activity. Based on these findings, it is possible to hypothesize that antiepileptic effects of NTS are related to alterations in neurotrophic factors levels, GABAergic neurotransmission and changes in properties and functions of the thalamo-cortical connection involved in neural plasticity in the developing brain.

## Conclusion

This study provides evidence that NTS slows down the development of absence epilepsy and comorbid depression-like symptoms in WAG/Rij rats that are genetically predisposed to absence epilepsy. The number and duration of absence seizures was reduced in both TS and DTP groups in comparison to control and MS groups. In addition, NTS in WAG/Rij rats resulted in an increased number of approaches to sucrose solution along with increased immobility latency in forced swimming test as compared with control and MS groups. TS also reduced immobility duration and increased active swimming time at the forced swimming test.

In conclusion, absence seizures and comorbid depression-like behaviors are sensitive to experimentally induced tactile stimulation. Therefore, we suggest that NTS might be used as a non-invasive and effective procedure for inhibition of epileptogenesis and depressive behaviors in absence epilepsy. Future studies in WAG/Rij rats may help to understand the mechanisms responsible for the modulating effects of NTS in this model.

## Data Availability Statement

The raw data supporting the conclusions of this article will be made available by the authors, without undue reservation.

## Ethics Statement

The animal study was reviewed and approved by the Ethics Committee of the University of Kocaeli, Turkey.

## Author Contributions

All authors listed have made a substantial, direct and intellectual contribution to the work, and approved it for publication.

## Conflict of Interest

The authors declare that the research was conducted in the absence of any commercial or financial relationships that could be construed as a potential conflict of interest.

## References

[B1] Aisa B. Tordera R. Lasheras B. Río J. D. Ramírez M. J. (2007). Cognitive impairment associated to HPA axis hyperactivity after maternal separation in rats. *Psychoneuroendocrinology* 32 256–266. 10.1016/j.psyneuen.2006.12.013 17307298

[B2] Antoniazzi C. T. Boufleur N. Pase C. S. Kuhn F. T. Dias V. T. Segat H. J. (2014). Tactile stimulation and neonatal isolation affect behavior and oxidative status linked to cocaine administration in young rats. *Behav. Processes* 103 297–305. 10.1016/j.beproc.2014.01.011 24468216

[B3] Antoniazzi C. T. Metz V. G. Roversi K. Freitas D. L. Vey L. T. Dias V. T. (2017). Tactile stimulation during different developmental periods modifies hippocampal BDNF and GR, affecting memory and behavior in adult rats. *Hippocampus* 27 210–220. 10.1002/hipo.22686 27874237

[B4] Ates N. Sahin D. Ilbay G. (2004). Theophylline, a methylxanthine derivative, suppresses absence epileptic seizures in WAG/Rij rats. *Epilepsy Behav.* 5 645–648. 10.1016/j.yebeh.2004.06.001 15380114

[B5] Bailey K. Im-Bolter N. (2018). Social context as a risk factor for psychopathology in children with epilepsy. *Seizure* 57 14–21. 10.1016/j.seizure.2018.03.007 29539588

[B6] Berghuis P. Dobszay M. B. Sousa K. M. Schulte G. Mager P. P. Härtig W. (2004). Brain-derived neurotrophic factor controls functional differentiation and microcircuit formation of selectively isolated fast-spiking GABAergic interneurons. *Eur. J. Neurosci.* 20 1290–1306. 10.1111/j.1460-9568.2004.03561.x 15341601

[B7] Blumenfeld H. Klein J. P. Schridde U. Vestal M. Rice T. Khera D. S. (2008). Early treatment suppresses the development of spike-wave epilepsy in a rat model. *Epilepsia* 49 400–409. 10.1111/j.1528-1167.2007.01458.x 18070091 PMC3143182

[B8] Boufleur N. Antoniazzi C. T. Pase C. S. Benvegnú D. M. Barcelos R. C. Dolci G. S. (2012). Neonatal tactile stimulation changes anxiety-like behavior and improves responsiveness of rats to diazepam. *Brain Res.* 20 50–59. 10.1016/j.brainres.2012.08.002 22898153

[B9] Boufleur N. Antoniazzi C. T. Pase C. S. Benvegnú D. M. Dias V. T. Segat H. J. (2013). Neonatal handling prevents anxiety-like symptoms in rats exposed to chronic mild stress: behavioral and oxidative parameters. *Stress* 16 321–330. 10.3109/10253890.2012.723075 22998434

[B10] Caldji C. Diorio J. Meaney M. J. (2003). Variations in maternal care alter GABA(A) receptor subunit expression in brain regions associated with fear. *Neuropsychopharmacology* 28 1950–1959. 10.1038/sj.npp.1300237 12888776

[B11] Caldji C. Francis D. Sharma S. Plotsky P. M. Meaney M. J. (2000). The effects of early rearing environment on the development of GABAA and central benzodiazepine receptor levels and novelty-induced fearfulness in the rat. *Neuropsychopharmacology.* 22 219–229. 10.1016/S0893-133X(99)00110-4 10693149

[B12] Castrén E. Rantamäki T. (2010). The role of BDNF and its receptors in depression and antidepressant drug action: reactivation of developmental plasticity. *Dev. Neurobiol.* 70 289–297. 10.1002/dneu.20758 20186711

[B13] Citraro R. Leo A. Santro M. D’agostino G. Constanti A. Russo E. (2017). Role of histone deacetylases (HDACs) in epilepsy and epileptogenesis. *Curr. Pharm. Des.* 23 5546–5562. 10.2174/1381612823666171024130001 29076408

[B14] Coenen A. M. van Luijtelaar E. L. (1987). The WAG/Rij rat model for absence epilepsy: age and sex factors. *Epilepsy Res.* 1 297–301. 10.1016/0920-1211(87)90005-2 3143552

[B15] Comeau W. L. Hastings E. Kolb B. (2007). Pre- and postnatal FGF-2 both facilitate recovery and alter cortical morphology following early medial prefrontal cortical injury. *Behav. Brain Res.* 180 18–27. 10.1016/j.bbr.2007.02.026 17408762

[B16] Cryan J. F. Lucki I. (2000). 5-HT4 receptors do not mediate the antidepressant-like behavioral effects of fluoxetine in a modified forced swim test. *Eur. J. Pharmacol.* 409 295–299. 10.1016/s0014-2999(00)00858-x 11108824

[B17] Czarnabay D. Dalmago J. Martins A. S. Queiroz A. Sperling L. E. Reis K. P. (2019). Repeated three-hour maternal deprivation as a model of early-life stress alters maternal behavior, olfactory learning and neural development. *Neurobiol. Learn. Mem.* 163:107040. 10.1016/j.nlm.2019.107040 31310813

[B18] Daskalakis N. P. Oitzl M. S. Schächinger H. Champagne D. L. de Kloet E. R. (2012). Testing the cumulative stress and mismatch hypotheses of psychopathology in a rat model of early-life adversity. *Physiol. Behav.* 106 707–721. 10.1016/j.physbeh.2012.01.015 22306534

[B19] Dobryakova Y. V. Dubynin V. A. van Luijtelaar G. (2008). Maternal behavior in a genetic animal model of absence epilepsy. *Acta Neurobiol. Exp.* 68 502–508. 10.55782/ane-2008-1716 19112473

[B20] Drinkenburg W. H. Coenen A. M. Vossen J. M. van Luijtelaar E. L. (1991). Spike-wave discharges and sleep-wake states in rats with absence epilepsy. *Epilepsy Res.* 9 218–224. 10.1016/0920-1211(91)90055-k 1743184

[B21] Fish E. W. Shahrokh D. Bagot R. Caldji C. Bredy T. Szyf M. (2004). Epigenetic programming of stress responses through variations in maternal care. *Ann. N. Y. Acad. Sci.* 1036 167–180. 10.1196/annals.1330.011 15817737

[B22] Freitas D. Antoniazzi C. T. Segat H. J. Metz V. G. Vey L. T. Barcelos R. C. (2015). Neonatal tactile stimulation decreases depression-like and anxiety-like behaviors and potentiates sertraline action in young rats. *Int. J. Dev. Neurosci.* 47(Pt B) 192–197. 10.1016/j.ijdevneu.2015.09.010 26449401

[B23] Giachino C. Canalia N. Capone F. Fasolo A. Alleva E. Riva M. A. (2007). Maternal deprivation and early handling affect density of calcium binding protein-containing neurons in selected brain regions and emotional behavior in periadolescent rats. *Neuroscience* 145 568–578. 10.1016/j.neuroscience.2006.12.042 17275195

[B24] Gibb R. L. Gonzalez C. L. Wegenast W. Kolb B. E. (2010). Tactile stimulation promotes motor recovery following cortical injury in adult rats. *Behav. Brain Res.* 214 102–107. 10.1016/j.bbr.2010.04.008 20394780

[B25] Giblin K. A. Blumenfeld H. (2010). Is epilepsy a preventable disorder? New evidence from animal models. *Neuroscientist* 16 253–275. 10.1177/1073858409354385 20479472 PMC2911489

[B26] Gonzalez A. Fleming A. S. (2002). Artificial rearing causes changes in maternal behavior and c-fos expression in juvenile femalerats. *Behav. Neurosci.* 116 999–1013. 10.1037//0735-7044.116.6.999 12492299

[B27] Gottmann K. Mittmann T. Lessmann V. (2009). BDNF signaling in the formation, maturation and plasticity of glutamatergic and GABAergic synapses. *Exp. Brain Res.* 199 203–234. 10.1007/s00221-009-1994-z 19777221

[B28] Grandin T. (1992). Calming effects of deep touch pressure in patients with autistic disorder, college students, and animals. *J. Child Adolesc. Psychopharmacol.* 2 63–72. 10.1089/cap.1992.2.63 19630623

[B29] Henneberger C. Jüttner R. Rothe T. Grantyn R. (2002). Postsynaptic action of BDNF on GABAergic synaptic transmission in the superficial layers of the mouse superior colliculus. *Neurophysiology* 88 595–603. 10.1152/jn.2002.88.2.595 12163512

[B30] Jutapakdeegul N. Casalotti S. O. Govitrapong P. Kotchabhakdi N. (2003). Postnatal touch stimulation acutely alters corticosterone levels and glucocorticoid receptor gene expression in the neonatal rat. *Dev. Neurosci.* 25 26–33. 10.1159/000071465 12876428

[B31] Kaffman A. Meaney M. J. (2007). Neurodevelopmental sequelae of postnatal maternal care in rodents: clinical and research implications of molecular insights. *J. Child. Psychol. Psychiatry.* 48 224–244. 10.1111/j.1469-7610.2007.01730.x 17355397

[B32] Karpova A. V. Bikbaev A. F. Coenen A. M. van Luijtelaar G. (2005). Morphometric Golgi study of cortical locations in WAG/Rij rats: the cortical focus theory. *Neurosci. Res.* 51 119–128. 10.1016/j.neures.2004.10.004 15681029

[B33] Karson A. Utkan T. Balcı F. Arıcıoðlu F. Ateş N. (2012). Age-dependent decline in learning and memory performances of WAG/Rij rat model of absence epilepsy. *Behav. Brain Funct.* 11:12. 10.1186/s12993-014-0052-6 25886443 PMC4375851

[B34] Kolb B. Gibb R. (2010). Tactile stimulation after frontal or parietal cortical injury in infant rats facilitates functional recovery and produces synaptic changes in adjacent cortex. *Behav. Brain Res.* 214 115–120. 10.1016/j.bbr.2010.04.024 20417237

[B35] Leal G. Afonso P. M. Salazar I. L. Duarte C. B. (2015). Regulation of hippocampal synaptic plasticity by BDNF. *Brain Res.* 1621 82–101. 10.1016/j.brainres.2014.10.019 25451089

[B36] Lipsky R. H. Marini A. M. (2007). Brain-derived neurotrophic factor in neuronal survival and behavior-related plasticity. *Ann. N. Y. Acad. Sci.* 1122 130–143. 10.1196/annals.1403.009 18077569

[B37] Lonstein J. S. Simmons D. A. Stern J. M. (1998). Functions of the caudal periaqueductal gray in lactating rats: kyphosis, lordosis, maternal aggression, and fearfulness. *Behav. Neurosci.* 112 1502–1518. 10.1037//0735-7044.112.6.1502 9926832

[B38] Lu B. (2003). BDNF and activity-dependent synaptic modulation. *Learn. Mem.* 10 86–98. 10.1101/lm.54603 12663747 PMC5479144

[B39] Lu B. Nagappan G. Lu Y. (2014). BDNF and synaptic plasticity, cognitive function, and dysfunction. *Handb. Exp. Pharmacol.* 220 223–250. 10.1007/978-3-642-45106-5_9 24668475

[B40] Lüttjohann A. van Luijtelaar G. (2015). Dynamics of networks during absence seizure’s on- and offset in rodents and man. *Front. Physiol.* 6:16. 10.3389/fphys.2015.00016 25698972 PMC4318340

[B41] Macbeth A. H. Stepp J. E. Lee H. J. Young W. S. III Caldwell H. K. (2010). Normal maternal behavior, but increased pup mortality, in conditional oxytocin receptor knockout females. *Behav. Neurosci.* 124 677–685. 10.1037/a0020799 20939667 PMC3175421

[B42] Martinowich K. Manji H. Lu B. (2007). New insights into BDNF function in depression and anxiety. *Nat. Neurosci.* 10 1089–1093. 10.1038/nn1971 17726474

[B43] Meis S. Endres T. Munsch T. Lessmann V. (2019). Impact of chronic BDNF depletion on GABAergic synaptic transmission in the lateral amygdala. *Int. J. Mol. Sci.* 3:E4310. 10.3390/ijms20174310 31484392 PMC6747405

[B44] Melo A. I. (2015). Role of sensory, social, and hormonal signals from the mother on the development of offspring. *Adv. Neurobiol.* 10 219–248. 10.1007/978-1-4939-1372-5_11 25287543

[B45] Mychasiuk R. Gibb R. Kolb B. (2013). Visualizing the effects of a positive early experience, tactile stimulation, on dendritic morphology and synaptic connectivity with Golgi-cox staining. *J. Vis. Exp.* 79:e50694. 10.3791/50694 24121525 PMC3935738

[B46] Nazish H. R. Ali N. Ullah S. (2018). The possible effect of SCN1A and SCN2A genetic variants on carbamazepine response among Khyber Pakhtunkhwa epileptic patients, Pakistan. *Ther. Clin. Risk Manag.* 14 2305–2313. 10.2147/TCRM.S180827 30538486 PMC6254658

[B47] Onat F. Y. van Luijtelaar G. Nehlig A. Snead O. C. III (2013). The involvement of limbic structures in typical and atypical absence epilepsy. *Epilepsy Res.* 103 111–123. 10.1016/j.eplepsyres.2012.08.008 22989853

[B48] Ovchinnikov A. Lüttjohann A. Hramov A. van Luijtelaar G. (2010). An algorithm for real-time detection of spike-wave discharges in rodents. *J. Neurosci. Methods* 194 172–178. 10.1016/j.jneumeth.2010.09.017 20933003

[B49] Popova N. K. Ilchibaeva T. V. Naumenko V. S. (2017). Neurotrophic factors (BDNF and GDNF) and the serotonergic system of the brain. *Biochemistry (Moscow)* 82 308–317. 10.1134/s0006297917030099 28320272

[B50] Raza S. Harker A. Richards S. Kolb B. Gibb R. (2015). Tactile stimulation improves neuroanatomical pathology but not behavior in rats prenatally exposed to valproic acid. *Behav. Brain Res.* 282 25–36. 10.1016/j.bbr.2014.12.055 25557797

[B51] Richards S. Mychasiuk R. Kolb B. Gibb R. (2012). Tactile stimulation during development alters behaviour and neuroanatomical organization of normal rats. *Behav. Brain Res.* 231 86–91. 10.1016/j.bbr.2012.02.043 22409973

[B52] Roversi K. de David Antoniazzi C. T. Milanesi L. H. Rosa H. Z. Kronbauer M. Rossato D. R. (2019). Tactile stimulation on adulthood modifies the HPA axis, neurotrophic factors, and GFAP signaling reverting depression-like behavior in female rats. *Mol. Neurobiol.* 56 6239–6250. 10.1007/s12035-019-1522-5 30741369

[B53] Russo E. Citraro R. Scicchitano F. De Fazio S. Perrotta I. Di Paola E. D. (2011). Effects of early long-term treatment with antiepileptic drugs on development of seizures and depressive-like behavior in a rat genetic absence epilepsy model. *Epilepsia* 52 1341–1350. 10.1111/j.1528-1167.2011.03112.x 21635238

[B54] Sarkisova K. van Luijtelaar G. (2011). The WAG/Rij strain: a genetic animal model of absence epilepsy with comorbidity of depression [corrected]. *Prog. Neuropsychopharmacol. Biol. Psychiatry* 35 854–876. 10.1016/j.pnpbp.2010.11.010 21093520

[B55] Sarkisova K. Y. Gabova A. V. (2018). Maternal care exerts disease-modifying effects on genetic absence epilepsy and comorbid depression. *Genes Brain Behav.* 17:e12477. 10.1111/gbb.12477 29604188

[B56] Sarkisova K. Y. Gabova A. V. Kulikov M. A. Fedosova E. A. Shatskova A. B. Morosov A. A. (2017). Rearing by foster Wistar mother with high level of maternal care counteacts the development of genetic absence epilepsy and comorbid depression in WAG/Rij rats. *Dokl. Biol. Sci.* 473 39–42. 10.1134/S0012496617020077 28508204

[B57] Sarkisova K. Y. Kulikov M. A. (2006). Behavioral characteristics of WAG/Rij rats susceptible and non-susceptible to audiogenic seizures. *Behav. Brain Res.* 166 9–18. 10.1016/j.bbr.2005.07.024 16183145

[B58] Sarkisova K. Y. Kuznetsova G. D. Kulikov M. A. van Luijtelaar G. (2010). Spike-wave discharges are necessary for the expression of behavioral depression-like symptoms. *Epilepsia* 51 146–160. 10.1111/j.1528-1167 19674046

[B59] Schridde U. Strauss U. Bräuer A. U. van Luijtelaar G. (2006). Environmental manipulations early in development alter seizure activity, Ih and HCN1 protein expression later in life. *Eur. J. Neurosci.* 23 3346–3358. 10.1111/j.1460-9568.2006.04865.x 16820024

[B60] Schridde U. van Luijtelaar G. (2004). The influence of strain and housing on two types of spike-wave discharges in rats. *Genes Brain Behav.* 3 1–7. 10.1111/j.1601-1848.2004.00034.x 14960010

[B61] Sitnikova E. (2011). Neonatal sensory deprivation promotes development of absence seizures in adult rats with genetic predisposition to epilepsy. *Brain Res.* 1377 109–118. 10.1016/j.brainres.2010.12.067 21194524

[B62] Sitnikova E. Rutskova E. M. Raevsky V. V. (2015). Reduction of epileptic spike-wave activity in WAG/Rij rats fostered by Wistar dams. *Brain Res.* 1594 305–309. 10.1016/j.brainres.2014.10.067 25449890

[B63] Sitnikova E. Rutskova E. M. Raevsky V. V. (2016). Maternal care affects EEG properties of spike-wave seizures (including pre- and post ictal periods) in adult WAG/Rij rats with genetic predisposition to absence epilepsy. *Brain Res. Bull.* 127:8491. 10.1016/j.brainresbull.2016.08.019 27593258

[B64] Smirnov K. Tsvetaeva D. Sitnikova E. (2018). Neonatal whisker trimming in WAG/Rij rat pups causes developmental delay, encourages maternal care and affects exploratory activity in adulthood. *Brain Res. Bull.* 140 120–131. 10.1016/j.brainresbull.2018.04.010 29684552

[B65] Smyk M. K. Coenen A. M. L. Lewandowski M. H. van Luijtelaar G. (2010). Endogenous rhythm of absence epilepsy: relationship with general motor activity and sleep-wake states. *Epilepsy Res.* 93 120–127. 10.1016/j.eplepsyres.2010 21146957

[B66] Tsybko A. S. Il’chibaeva T. V. Kondaurova E. M. Bazovkina D. V. Naumenko V. S. (2014). Effect of central administration of the neurotrophic factors BDNF and GDNF on the functional activity and expression of 5-HT2A serotonin receptors in mice genetically predisposed to depressive-like behavior. *Mol. Biology* 48 864–869. 10.1134/s0026893314060181 25845239

[B67] van Luijtelaar E. L. Coenen A. M. (1986). Two types of electrocortical paroxysms in an inbred strain of rats. *Neurosci Lett.* 70 393–397. 10.1016/0304-3940(86)90586-0 3095713

[B68] van Luijtelaar E. L. Coenen A. M. (1988). Circadian rhythmicity in absence epilepsy in rats. *Epilepsy Res.* 2 331–336. 10.1016/0920-1211(88)90042-3 3143564

[B69] van Luijtelaar G. Budziszewska B. Jaworska-Feil L. Ellis J. Coenen A. Lasoń W. (2001). The ovarian hormones and absence epilepsy: a long-term eeg study and pharmacological effects in a genetic absence epilepsy model. *Epilepsy Res.* 46 225–239. 10.1016/s0920-1211(01)00277-7 11518624

[B70] van Luijtelaar G. Sitnikova E. (2006). Global and focal aspects of absence epilepsy: the contribution of genetic models. *Neurosci. Biobehav. Rev.* 30 983–1003. 10.1016/j.neubiorev.2006.03.002 16725200

[B71] van Luijtelaar G. Zobeiri M. (2014). Progress and outlooks in a genetic absence epilepsy model (WAG/Rij). *Curr. Med. Chem.* 21 704–721. 10.2174/0929867320666131119152913 24251564

[B72] van Vugt R. W. Meyer F. van Hulten J. A. Vernooij J. Cools A. R. Verheij M. M. (2014). Maternal care affects the phenotype of a rat model for schizophrenia. *Front. Behav. Neurosci.* 8:268. 10.3389/fnbeh.2014.00268 25157221 PMC4128220

[B73] White H. S. Löscher W. (2014). Searching for the ideal antiepileptogenic agent in experimental models: single treatment versus combinatorial treatment strategies. *Neurotherapeutics* 11 373–384. 10.1007/s13311-013-0250-1 24425186 PMC3996126

[B74] Yamada K. Mizuno M. Nabeshima T. (2003). Role for brain-derived neurotrophic factor in learning and memory. *J. Pharmacol. Sci.* 91 267–270. 10.1016/S0024-3205(01)01461-8 12719654

[B75] Yoshii A. Constantine-Paton M. (2010). Postsynaptic BDNF-TrkB signaling in synapse maturation, plasticity, and disease. *Dev. Neurobiol.* 70 304–322. 10.1002/dneu.20765 20186705 PMC2923204

[B76] Zhu G. Sun X. Yang Y. Du Y. Lin Y. Xiang J. (2019). Reduction of BDNF results in GABAergic neuroplasticity dysfunction and contributes to late-life anxiety disorder. *Behav. Neurosci.* 133 212–224. 10.1037/bne0000301 30714802

